# How Will the Future of Work Shape OSH Research and Practice? A Workshop Summary

**DOI:** 10.3390/ijerph18115696

**Published:** 2021-05-26

**Authors:** Sarah A. Felknor, Jessica M. K. Streit, Michelle McDaniel, Paul A. Schulte, L. Casey Chosewood, George L. Delclos, on behalf of the Workshop Presenters and Participants

**Affiliations:** 1 National Institute for Occupational Safety and Health, Atlanta, GA 30333, USA; LChosewood@cdc.gov; 2 National Institute for Occupational Safety and Health, Cincinnati, OH 45226, USA; JStreit@cdc.gov (J.M.K.S.); PSchulte@cdc.gov (P.A.S.); 3 Southwest Center for Occupational and Environmental Health, The University of Texas Health Science Center at Houston School of Public Health, Houston, TX 77030, USA; Michelle.R.McDaniel@uth.tmc.edu (M.M.); George.Delclos@uth.tmc.edu (G.L.D.)

**Keywords:** expanding occupational safety and health paradigm, future of work, research methods, personal and socioeconomic risk factors, worker well-being, working-life continuum

## Abstract

Growth of the information economy and globalization of labor markets will be marked by exponential growth in emerging technologies that will cause considerable disruption of the social and economic sectors that drive the global job market. These disruptions will alter the way we work, where we work, and will be further affected by the changing demographic characteristics and level of training of the available workforce. These changes will likely result in scenarios where existing workplace hazards are exacerbated and new hazards with unknown health effects are created. The pace of these changes heralds an urgent need for a proactive approach to understand the potential effects new and emerging workplace hazards will have on worker health, safety, and well-being. As employers increasingly rely on non-standard work arrangements, research is needed to better understand the work organization and employment models that best support decent work and improved worker health, safety, and well-being. This need has been made more acute by the SARS-CoV-2 global pandemic that has resulted in dramatic changes in employment patterns, millions of lost jobs, an erosion of many economic sectors, and widespread disparities which further challenge occupational safety and health (OSH) systems to ensure a healthy and productive workplace. To help identify new research approaches to address OSH challenges in the future, a virtual workshop was organized in June 2020 with leading experts in the fields of OSH, well-being, research methods, mental health, economics, and life-course analysis. A paradigm shift will be needed for OSH research in the future of work that embraces key stakeholders and thinks differently about research that will improve lives of workers and enhance enterprise success. A more transdisciplinary approach to research will be needed that integrates the skills of traditional and non-traditional OSH research disciplines, as well as broader research methods that support the transdisciplinary character of an expanded OSH paradigm. This article provides a summary of the presentations, discussion, and recommendations that will inform the agenda of the Expanded Focus for Occupational Safety and Health (Ex4OSH) International Conference, planned for December 2021.

## 1. Introduction

There is a growing body of literature to demonstrate that the future of work will have increasingly profound effects on the jobs that workers around the world perform every day [1,2,3,4,5,6,7]. These changes will affect not only the nature of work but also the workplace and the workforce, and existing paradigms of worker health, safety, and well-being will need to shift to meet new challenges for worker protections [8,9,10,11]. Many of these changes will be heralded by technological advances such as robotics and artificial intelligence (AI) that have already arrived in the workplace and are expected to displace a significant number of jobs in the U.S. and Europe, at least in the short term [12,13,14,15].

New work arrangements may further blur work-life boundaries, by extending the workplace into home life and into added hours of employment. This will require a more holistic view of worker health, safety, and well-being in the context of work and non-work hazards [16]. The global pandemic of 2020 has greatly accelerated many of the changes in the nature of work that were well underway, and the resulting economic downturn will likely extend the length of time that older people remain in the workforce, out of financial necessity [17]. Coupled with increases in life expectancy around the globe that have resulted in people working beyond traditional retirement age, these factors will contribute to a working-life continuum that includes post-retirement jobs [18].

**Figure 1:**
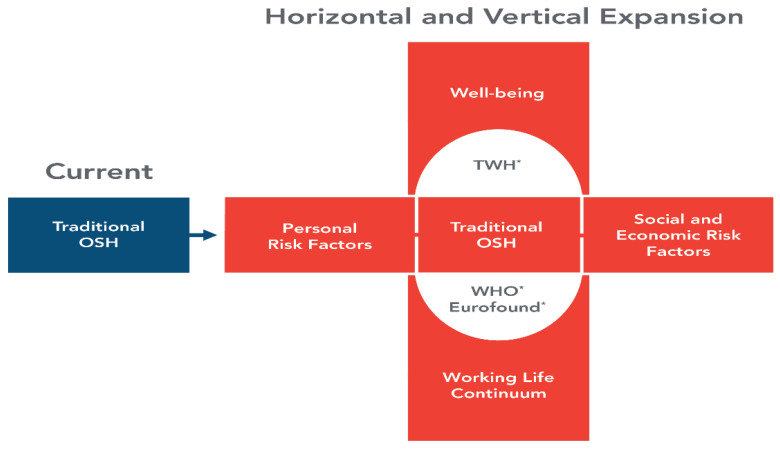
An expanded focus for occupational safety and health [11]. * Horizontal and vertical expansion build on the work of WHO [22], Eurofound [23], and *Total Worker Health* (TWH) [24,25].

The need for an expanded focus for occupational safety and health (OSH) to prepare for such changes anticipated in the future of work has been well documented [8,11,16,19,20,21]. One model for an expanded focus for OSH (Figure 1) calls for broadening the traditional OSH domains, which previously focused on evaluating workplace exposures and hazards and developing risk-prevention strategies. This expanded model considers the complex interaction of work and non-work factors that influence worker safety, health, and well-being [11]. The horizontal expansion includes personal, social, and economic factors; and the vertical expansion includes measurement of risk extended to the working-life continuum that includes worker well-being as an outcome [8,11]. This model builds on previous work of the World Health Organization (WHO), European Foundation for the Improvement of Living and Working Conditions (Eurofound), and the NIOSH *Total Worker Health* ^®^ program (TWH) [22,23,24,25], and considers a broader set of work-life and well-being factors that support the United Nations 2030 Agenda for Sustainable Development Goals calling for sustained economic growth and decent work for all nations [26].

**Figure 2:**
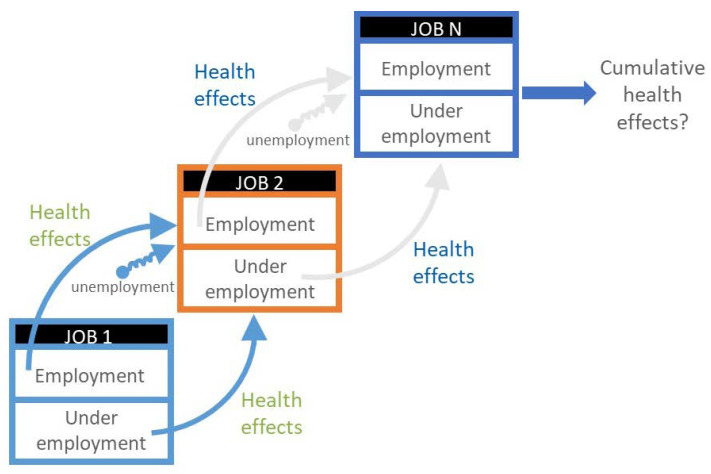
Model of health burden along the working-life continuum [11].

Under this expanded model, it is no longer sufficient to restrict OSH to workplace confined factors affecting worker health and safety, and work-related illness and injury. OSH can no longer be separated from personal health and well-being because both are inextricably linked [27]. Instead, the multifaceted well-being effects of work and non-work exposures should be considered in concert and within the broader context of macro-level social and economic factors that affect health, safety, and well-being over the working-life continuum from pre-work to post-work. The OSH field will need to adapt to changes in the nature of work through an expanded focus that includes well-being and the working-life continuum. A model for how OSH might consider health effects, positive or adverse, along the working-life continuum is shown in Figure 2 [11]. This model shows a potential working-life continuum that not only considers the well-established health effects that may result from employment, but also the understudied health effects of periods of unemployment or under-employment (not enough paid work or working below a person’s skill and abilities). There is research to suggest that periods of unemployment and underemployment may lead to mental health effects, increased odds of poor health or new health conditions, premature mortality, and low levels of well-being [28,29,30]. The model in Figure 2 may guide new research approaches needed to expand our understanding of, and preparedness for, OSH challenges in the future.

To facilitate dialogue around the topic of new research approaches to support an expanded focus for OSH in the changing work environment, the Southwest Center for Occupational and Environmental Health at UTHealth School of Public Health and the National Institute for Occupational Safety and Health (NIOSH) convened a virtual workshop in June 2020. This workshop was one activity under a three-year Cooperative Agreement (Grant U13OH011870), which has already been described [8].

## 2. Materials and Methods

The objectives of the workshop were to: (a) examine how the future of work will impact OSH research; (b) identify gaps and needs in OSH research; and (c) inform the agenda of a larger international conference, to be held in 2021. The workshop was organized to address the horizontal and vertical expansion of the expanded OSH model described in Figure 1. Due to the public health threat of the COVID-19 pandemic and its associated travel restrictions, the workshop was convened using a web-based platform. The workshop discussions of the two themes were held on separate days. Pre-recorded presentations were available to participants for on-demand viewing one week prior to each live discussion date. The workshop agenda is provided in Appendix A.

The Organizing Committee selected workshop participants using a modified snowball technique where subject matter experts were asked to recommend participants based on their knowledge or practice in a specific topic area relevant to the major themes of the workshop. The organizers also considered diversity and inclusion criteria to provide a broad range of views. The virtual nature of the workshop permitted a total of 53 participants (which included workshop moderators) from the U.S. and European countries to attend, with substantial overlap between the two sessions: 45 participants and 3 workshop moderators attended the Theme 1 session on June 4, and 40 participants and 3 workshop moderators attended the Theme 2 session on June 25. Participants were leaders in OSH research, academia, government, industry, and labor. A list of workshop speakers, participants, and Organizing Committee members is provided in Appendix B.

Speakers were identified based on their expertise in topic areas related to OSH research needs, methods, and by experience with key constructs in the horizontal and vertical expansions. These constructs included traditional and non-traditional risk factors (e.g., workplace exposures vs. non-work risk factors that may influence or exacerbate workplace exposures), working-life continuum, and well-being. The presentations were designed to elicit focused discussion between workshop participants during the live facilitated session. Each workshop session was preceded by a recorded keynote address discussing key thematic issues. These were followed by a set of shorter talks that addressed a particular topic or sub-topic within the overall theme.

Introductory addresses by NIOSH thought leaders envisioned how the future of work might impact OSH research and training needs and provided background and rationale for the model for an expanded focus for OSH (Figure 1). These pre-recorded presentations [31,32], which provided a key context for the two workshop themes, have been previously summarized [8].

### 2.1. Theme 1: The Horizontal Expansion of OSH

The live virtual discussion of Theme 1 was organized to consider three research domains pertinent to an expanded focus of risk factors anticipated in future conditions: (1) research needs, (2) research methods, and (3) research translation (how research is applied in real world settings). Prior to the live session, participants were asked to view the recorded keynote and short presentations related to the keynote theme, summarized below.

#### 2.1.1. Horizontal Expansion of OSH Presentations

As the focus of OSH expands to include personal, social, and economic risk factors, research on worker safety, health, and well-being must examine the complex interrelationships of work and other life domains [33]. This will require the development and adoption of more comprehensive conceptual models for OSH, such as that from Sorensen et al. [34], which consider the social, technological, economic, environmental, and political (STEEP) context; employment and labor patterns; and enterprise- and worker-level factors. These models should be designed to represent an amalgam of perspectives and interests in order to create a common vision, shared principles, and standardized lexicon for a transdisciplinary OSH community [33]. However, the models must also remain agile enough to recognize and incorporate nontraditional occupational hazards. For example, livable wages are a fundamental dimension of job quality, supporting the inclusion of low wages as a critical job hazard within the horizontal expansion [35,36]. Similarly, work-life integration challenges represent another job hazard for the horizontal expansion. Difficulties integrating the work and personal domains of life are associated with poor health outcomes, including cardiovascular disease, substance use disorders, and poor mental health; negative outcomes at home, such as familial conflict, marital stress, and decreased satisfaction; and adverse work outcomes, including absenteeism, turnover, reduced employee engagement, and performance decrements [37].

Transitioning to a broader set of worker risk categories will have implications for OSH research priorities, methods, and translation. At a fundamental level, OSH literature reviews will need to expand to include both occupational and non-occupational sources to adequately capture occupational and personal risk factors and their cumulative effects [38]. Algorithms are available to assist with the identification of potential hazards and theorized exposure-outcome associations [39]. Multilevel modeling is a useful tool for analyzing hazardous events within the societal and cultural contexts in which they occur [40,41,42]. Latent class analysis can help to identify profiles of workers based on their exposures [42]. As intervention designs adapt to target multiple outcomes and address multi-level exposure inequalities, additional investment in translation research can elucidate the relationship between research outputs and downstream outcomes [43,44]. Collectively, these changes will improve the OSH community’s ability to understand how emerging issues might affect the future workforce, its capacity to mitigate their potential negative effects or harness their positive ones.

#### 2.1.2. Horizontal Expansion of OSH Participant Discussion Groups

At the start of the live session on June 4, participants selected discussion questions from among a list of probing questions relevant to each of the three domains of Theme 1 (research needs, research methods, and research translation) developed by the organizers (Appendix C). Participants completed an online poll to vote for the top three most important questions under each domain. The question with the most votes under each domain was selected and discussed in breakout groups.

Participants were then randomly assigned in roughly equal number to three facilitated virtual breakout sessions (13–15 participants per breakout), and each breakout group discussed one of the top three probing questions. Moderators led the breakout group discussions following a previously designed discussion guide. Each group was asked to identify the following critical components for each question: (a) identity of key stakeholders; (b) additional resources or information needed to address the domain; (c) desired outcomes for each domain; and (d) next steps and remaining gaps.

The breakout sessions were facilitated by members of the Organizing Committee (S.A.F., G.L.D., J.M.K.S.). In each breakout group, an assigned notetaker compiled summary results. The facilitators presented these summaries to the larger participant group at the conclusion of the session. The results from the workshop discussion of Theme 1 are presented Section 3.1 below.

### 2.2. Theme 2: The Vertical Expansion of OSH

The same methods were employed for the second session of the workshop on June 25. Participants were asked to view the recorded keynote and short presentations related to the keynote theme, summarized below.

#### 2.2.1. Vertical Expansion of OSH Presentations

Worker well-being is a key outcome in the vertical expansion of OSH, and mental health is a critical component of worker well-being. Workers’ mental health can be adversely impacted by job design factors, such as having time pressures or too much/little work; role issues, including role ambiguity or conflict; poor relationships with supervisors, colleagues, or subordinates; job insecurity and uncertainty; and negative organizational structure and climate [45]. Conversely, secure, meaningful work with supportive supervisors and colleagues can dramatically enhance health opportunities at work and beyond. Investigations of mental health and worker well-being require the use of both subjective and objective data [46]. Today, a growing body of evidence from the UK presents a compelling business case for investing in workers’ mental health [45,47,48,49,50]. Future research in the vertical expansion can build upon this evidence base to help position workplace mental health and well-being as strategic business issues.

To accurately assess mental health and other well-being outcomes, OSH research must adopt a working-life continuum perspective (i.e., analyzing workers across the lifespan) to account for the fact that worker health does not simply begin with the first paycheck [51,52]. Rather, there are critical or sensitive developmental periods, transitions, and accumulation of risk through sequences of linked events or exposures occurring from birth onward that have lasting effects throughout the life course [53,54]. The nature and timing of these events and transitions are constructed by the underlying social context, which is itself shaped by political changes and the global economy [52]. Furthermore, it is important to recognize that the health effects of work continue after work ends. Job complexity, autonomy, feedback, task variety, learning opportunities, and work stress are all job characteristics known to impact cognitive functioning, which is a key aspect for successful aging [55]. Indeed, along with changes in physical abilities and quality of life, changes in cognitive function represent one of the key measures of health and well-being in retirement and beyond [56]. Within the vertical expansion, new OSH models must acknowledge and account for these multi-level life course factors.

#### 2.2.2. Vertical Expansion of OSH Participant Discussion Groups

Participants were given a list of probing questions, inspired by the Theme 2 presentations, and organized into two topic areas: (1) working-life continuum and (2) well-being research. Participants were polled to select the top three most pressing and important questions under each topic. Attendees were randomly assigned to three breakout sessions, and each breakout considered different questions related to the working-life continuum and well-being research.

As with the first workshop session, the breakout sessions from Theme 2 were facilitated by Organizing Committee members (S.A.F., G.L.D., J.M.K.S.), and notetakers compiled summary results that were presented by the facilitators to the larger participant group at the conclusion of the session. The results from the workshop discussion of Theme 2 are presented in Section 3.2 below.

## 3. Results and Discussion

### 3.1. Theme 1: The Horizontal Expansion of OSH Breakout Group Discussion

Workshop participants selected the following questions to guide their discussions of the horizontal expansion: (1) How do we build the evidence base for the interrelationships between work and other life domains to support the expansion of OSH paradigms? (research needs); (2) Given the changing paradigms anticipated in the future of work, what new methods will be needed to fully explore research questions? (research methods); (3) What new approaches are needed to apply research findings in the future of work? (research translation). Each workshop breakout group met to identify key stakeholders, resources and information needed, desired outcomes, and remaining gaps associated with one of these areas. The results of the breakout group discussions were combined for each key element (stakeholders, resources, outcomes, and gaps) and the data are summarized in Table 1.

While there was general agreement of who the key OSH stakeholders are, new communication methods and approaches are needed to reach a wide range of groups, as are specialized messages, especially when communicating research results to workers. New resources and information needed in the future of OSH research include establishing new connections with traditional and non-traditional researchers where resources could be shared, as well as finding new research methods that would build research capacity across interprofessional and transdisciplinary lines. Desired outcomes for OSH research in the future of work included conducting research that contributes to changes in policy and practices and improving existing OSH systems to reflect the actual demographics of society. OSH research that considers worker health, safety, and well-being over the working life course is needed, and expanding OSH research concepts to include equity and inclusion issues will be important in the future. Research that optimizes worker and organizational well-being was identified as a priority outcome, and the need to incorporate research translation activities into research design will be important to the future of OSH research. Several gaps were identified that centered around the need for and access to data that incorporates information on wages, productivity, workplace policies, non-standard work arrangements, and small businesses. Gaps that resonated with resources and desired outcomes included the need to incorporate a life course approach to OSH research along with new research methods to support those aims.

### 3.2. Theme 2: The Vertical Expansion of OSH Breakout Group Discussions

Workshop participants selected the following as the top three questions related to a working-life continuum perspective in OSH research: (1.a.) How do we engage occupational safety and health researchers to think differently about occupational safety and health? What new research methods are needed in the expanded focus?; (2.a.) How can we stimulate research to quantify work-life exposures that promotes post-retirement health and well-being throughout the stakeholders which engage in this type of work?; and (3.a.) From a life course perspective, critical events and transitions occurring in all pre-work years, including childhood and adolescence, can have lasting impacts on worker health and well-being. How do we increase occupational safety and health interest in pre-work research? Further, how do we engage “pre-workers” in occupational safety and health research and translation?

The data from the working-life continuum breakout group discussions are provided in Table 2 and briefly summarized here. Question 1.a. discussion identified the need for stakeholder evidence to lead researchers to think differently about OSH. Clearly defined outcomes and funding to support new types of research will be critical to moving the field. Consumers of OSH research will need to balance the desire for quick results with the benefits of working life-course research that supports life transitions, and researchers will need training in new methods related to working life-course inquiry. And making research results more accessible to key stakeholders, including workers and employers, will be needed to facilitate research translation efforts. Question 2.a. discussion focused on expanding the OSH research paradigm to include pre-workers, typically young people who have not joined the workforce in any capacity. A key discussion centered on the need for an evidence base of interventions in early childhood which eventually have work-related or labor market implications once those children join the workforce. Research methods that consider multiple employers over a lifetime and common definitions of terms among OSH researchers will also be needed. And discussion of question 3.a. identified the need to involve key retiree stakeholder groups such as AARP (formerly known as the American Association of Retired Persons) and unions to support expanded research into how working life exposures can promote post-retirement health and well-being. A new set of research methods will be needed to explore how work can have a beneficial impact on post-working life well-being. Workshop participants selected the following as the top three questions related to well-being research in OSH: (1.b.) How can well-being be used effectively in risk assessment and policy development? (2.b.) How do we expand the OSH community adoption of common measures and metrics that allow us to make ‘apples-to-apples’ comparisons of well-being indicators and influence decision-making at the individual, business, industry, sector, and policy levels? and (3.b.) How we can incentivize companies to invest in practices, programs, and policies that positively impact workers’ mental health? Each breakout group considered research needs, research methods, and research translation associated with one of the questions.

The data from the well-being research in OSH breakout group discussions are provided in Table 2 and briefly summarized here. Question 1.b. was central to the premise of well-being as a primary outcome for workers. To achieve this, OSH researchers will need a common set of definitions for well-being outcomes to promote a unified understanding of well-being, how it can be supported by workplace policies and practices, and how it can be measured in a systematic way. Incorporating well-being into OSH research provides an opportunity to collaborate with other disciplines and stakeholders that may not have been involved in OSH related issues previously. This would help communicate the value of well-being to policymakers and employers. Question 2b. considered how we can expand the OSH measures and metrics to make accurate comparisons of well-being indicators at the business and policy level. In agreement with discussion of question 1.b. above, the need for standardized metrics and common understanding of well-being will be central to this effort. To achieve this, there will need to be broad consideration of work and non-work factors as well as types of employment across multiple worker populations. And discussion of question 3.b. focused on approaches to incentivize companies to invest in practices and programs that have beneficial effects on workers’ mental health. There was general agreement that the need to reduce the stigma associated with mental health issues would be critical. Additional training in the OSH curriculum will be needed along with more effective interventions and improved evaluation research to identify successful outcomes. Discussants identified the need for OSH to make a strong business case for the connection between mental health and employer outcomes of interest. Finally, targeted messaging at the organizational and policymaker levels that promotes recognition of mental health issues and psychosocial exposures as job hazards and outcomes is needed.

## 4. Conclusions and Recommendations

The following recommendations—distilled from workshop discussions—are offered to help achieve an expanded focus for OSH in the future of work. The concepts of the expanded focus are still somewhat formative and the distinction between the horizontal and vertical expansion is not implicitly understood. While much of the discussion relates to social behavioral and epidemiologic research, the OSH field should not abandon evolving traditional research methods, such as toxicology, that are developing in response to new materials that are not well studied.

The expanded challenges described in this paper must also be considered against a backdrop of limited funds for OSH in many countries. Stretching these funds to address this expanse will require understanding of the most important threats to worker well-being and what the OSH community can actually do about them. Recruiting additional stakeholders and sources of funding will be critical to this expanded effort.

### 4.1. Stakeholders

There is a need for key stakeholders to think differently about the future of OSH and commit to research that will both improve the lives of workers and enhance the success of enterprises. One of the critical elements of new OSH research paradigms is stakeholder involvement. The stakeholder groups included in OSH research will need to go beyond the traditional discipline-specific groups and associations that have long supported the field. OSH is a multifaceted field that requires stakeholders to move research into practice. Stakeholders will be needed to provide balanced representation of the labor perspective and expertise that will be central to the future of work. The OSH field may benefit from adoption of a systematic approach to stakeholder identification to strengthen and support the development of key partners who will be critical to an expanded focus for OSH in the future.

### 4.2. Research Approach

A more transdisciplinary approach is needed in OSH research of the future. To achieve this, both traditional and non-traditional OSH researchers will be needed to address the myriad factors workers will face. Traditional OSH researchers represent the well-known OSH domains of occupational safety, industrial hygiene, occupational medicine, occupational health nursing, ergonomics, health physics, injury prevention, epidemiology, and occupational health psychology. The non-traditional OSH researchers in an expanded focus for OSH would be more systems-based and interprofessional to include disciplines new to OSH such as anthropology, applied economics, architecture, climate science, education, human relations, political science, sociology, and urban planning [11]. Along with work-related factors, these will include both non-work factors and STEEP contextual factors. Research will be needed to inform workplace policy actions that support worker well-being and productivity and establish a basis for conducting research across the working-life continuum to better understand the connection between work and well-being, even into retirement. As non-standard work arrangements (which are characterized by the temporary nature of work such as short-term contracts or gig work and the erosion of workplace protections) are expected to increase significantly, OSH research must include approaches that can access workers in these settings [57]. Related to this will be research that improves understanding of how precarious, low-wage, dead-end jobs are created and what work organization and employment models best support decent work and improved worker health, safety, and well-being [26,58].

### 4.3. Research Methods

Different research methods are needed to support the transdisciplinary character of the expanded paradigm of OSH in the future. Researchers will need to balance the cost benefit of traditional cohort studies over the working-life continuum with the need for shorter-term results that can improve worker health, safety, and well-being outcomes. Future OSH methods should consider work transitions over the life course, including the health effects of periods of unemployment or underemployment. The OSH research paradigm must shift away from a one-employer model and consider that most people will have multiple employers in their working lives. Research methods should assess the economics of workplace injury and illness over the life course and continue to consider the broader societal costs of work-related outcomes that continue into retirement. The new transdisciplinary OSH research community will need a commonly held lexicon that integrates more macro-level STEEP factors, multiple employers, and organizational dynamics rather than individual-level etiologic studies of the past.

### 4.4. Additional Recommendations

Recommendations for working-life continuum studies should not obscure the need for answers to OSH challenges now as well as in the future. Researchers will need to manage the competing demands of the push for more immediate results with the pull of longer-term benefit of studies that consider the life course continuum.

There is a challenge in the U.S. with no centralized and accessible repository for work history or medical data, making working-life continuum research difficult. Several European countries have systems in place where workers and healthcare providers can access work history data at any point in time [59]. The European Framework for Action on Integrated Health Services Delivery describes a health services delivery system that provides comprehensive services over the life-course and promotes well-being through a transdisciplinary approach [60]. There will be a number of cultural constraints to overcome in the U.S. before such an integrated system could be in place, including concerns over privacy and confidentiality, but research that assesses feasibility of such a system in the U.S. could be of great potential benefit to research over the working-life continuum.

The important role of work organization on the health, safety, and well-being of workers must not be overlooked. One such example is the lack of OSH attention to repetitive or monotonous work, which produces apathy and alienation instead of well-being and is considered to be a risk factor for adverse physical and mental health outcomes [61,62,63,64]. Research into alternative work design can contribute to better health outcomes. The NIOSH Healthy Work Design and Well-being Program has developed a research agenda that includes an emphasis on optimizing work arrangements [65]. Gaps and research questions have been identified in this area, and they might be a place for starting to address alternative work design.

These recommendations will inform the agenda for the Expanded Focus for Occupational Safety and Health (Ex4OSH) International Conference scheduled to take place in Houston, Texas, USA in December 2021. They reflect the collective input of the workshop participants who are subject matter experts in topic areas that pertain to the workshop objectives. They may not necessarily represent the views of other disciplines or worker populations.

## Figures and Tables

**Table 1:** Summary of Key Elements in the Horizontal Expansion.

Stakeholders	Resources or Information	Desired Outcome	Remaining Gaps
Include multiple stakeholder groups from government, private sector, OSH professions, labor and employers Consider how we communicate results to stakeholders at each level, varying content and style as appropriate Place special emphasis on communicating results to workers, especially younger workers, new workers, and the unemployed	Establish mutual understanding of connections between non-OSH and traditional OSH researchers Identify pathways to combine methods—interprofessional and transdisciplinary Incorporate behavioral methods at beginning of study Go beyond studying workers only in the workplace Better integrate environmental variables Look for high-quality big data	Conduct research today that is relevant for 5–10 years Conduct research that contributes to changes in policy, implementation, and behaviors Improve existing OSH systems by modifying variables to reflect societal perspective (e.g., sex/gender, race/ethnicity) Design studies to better follow workers across jobs and life course Expand OSH concepts (e.g., role of power dynamics or equity/justice in shaping change) Optimize shared benefits of worker and organizational well-being Refine the framework for OSH—what is included? What is not included? Design research studies that include translation and marketing plans from their inception	Seamlessly connect data between work and other life domains Incorporate variables related to wages, productivity measures, leave and benefits policies, nonstandard work arrangements Collaborate with smaller employers and stakeholder groups Incorporate life course approach to OSH research, including cumulative risk assessment, participatory and qualitative methods, and impact evaluation Shorten the lag time between study design and implementation Design studies that are more generalizable, adoptable, adaptable, and sustainable, even in the face of uncertainty Increase understanding and inclusion of nonstandard workplace (e.g., temporary work, gig or platform work, informal work, multiple jobs, etc.) Move beyond randomized controlled trials (RCTs) to include other approaches (e.g., natural/observational studies and analyses of archival data) Find most effective ways to leverage intermediate outcomes to demonstrate progressive change Explore the inputs/antecedents that result in the creation of ‘bad jobs’ and the changes that are needed to improve employment and organizational models Build capacity to conduct shorter-term research that provides real-time insights to the rapid changes we see daily

**Table 2:** (a) Summary of Needs, Methods, and Translation in Working-Life Continuum. (b) Summary of Needs, Methods, and Translation in Well-being Research.

**a. Summary of Needs, Methods, and Translation in Working-Life Continuum**			
**Question 1.a.**	**Research Needs**	**Research Methods**	**Research Translation**
How do we engage occupational safety and health researchers to think differently about occupational safety and health? What new research methods are needed in the expanded focus?	Documented evidence of stakeholder support, especially among workers Clearly defined outcomes of interest Funding mechanisms that support this type of research Collaboration and cooperation among employers, government sources, and researchers for data sharing	Balance desire for cohort studies over the working-life course with need to provide shorter term results Focus on the different impacts of life course transitions at different life stages (e.g., being unemployed right out of school vs. unemployed after age 50) Training in life course research methods	Make research results more accessible, in particular the cost/benefit data on sickness absence, healthcare, or availability of paid sick leave
**Question 2.a.**	**Research Needs**	**Research Methods**	
How do we increase occupational safety and health interest in pre-work research? And how do we engage ‘pre-workers’ in occupational safety and health research and translation?	Established policies and workplace interventions (e.g., childcare, paid leave) that support parents in rearing children Exploration of parenting and care-giving roles in the context of work Evidence base for interventions in early childhood which eventually have work-related or labor market implications Increased OSH researchers’ interest in areas that have impact years later	Research methods that consider multiple employers over a lifetime Commonly held definitions of what the pre-work period is	
**Question 3.a.**	**Research Needs**	**Research Methods**	**Research Translation**
How can we stimulate research to quantify work-life exposures that promote post-retirement health and well-being?	Stakeholder involvement to identify retirees (e.g., AARP, unions, healthcare systems, public health organizations and agencies, Social Security Administration) Common definition of ‘healthy retirement’	Methods that consider very different retirement experiences, as retirement is not a single construct that is the same for everyone Approaches to identify exposures that affect health and well-being during and after work in retirement Large-scale cohort-based surveillance of work and nonwork exposures Methods that consider who bears the cost of ill health in retirement because of exposures over a working-life course	Evidence that supports stakeholder awareness of links between health and work and health in retirement
**b. Summary of Needs, Methods, and Translation in Well-being Research.**			
**Question 1.b.**	**Research Needs**	**Research Methods**	**Research Translation**
How can well-being be used effectively in risk assessment and policy development?	Common definitions for relevant well-being outcomes Better definitions of life course exposures, recognizing that the curve is not smooth Standardized metrics (metricize, monetize, create value) for well-being Studies that leverage challenging issues at national level around well-being that are not strictly OSH-related but that may impact productivity and other OSH measures	Studies that build upon opportunities arising from the SARS-CoV-2 pandemic to investigate mental health issues for workers (e.g., mental health effects of new forms of work, in a quicker timeline) Revised approaches to risk assessment Statistical analysis methods in life course research, including latent trajectory or structural equation techniques Better understanding of how the different paths influencing latent trajectory converge over the life course and link them clearly to life course outcomes	Communication of the value of well-being to policymakers Strategies that link well-being measures to new policies
**Question 2.b.**	**Research Needs**	**Research Methods**	**Research Translation**
How do we expand the OSH community adoption of measures and metrics, for example, econometrics, that allow us to make ‘apples-to-apples’ comparisons of well-being indicators and influence decision-making at the individual business industry sector and policy levels?	Standard agreement on well-being indicators, to facilitate discussions of ‘apples-to-apples’ comparisons (which are currently very challenging) Broad cross-sections of workers considered in well-being indicators	Agreed-upon well-being measures Broad consideration of work and non-work factors Consideration of type of employment, (i.e., s multiple employers or self-employment, gig work) across a spectrum of worker populations	
**Question 3.b.**	**Research Needs**	**Research Methods**	**Research Translation**
How we can incentivize companies to invest in practices, programs, and policies that positively impact workers’ mental health?	Increased awareness of and reduced stigma associated with mental health conditions Additional training of OSH community and industry to recognize mental health as a leading workplace issue More effective interventions More evaluation research Establish the true burden of mental health issues More best practices and success stories Better understanding of the challenges employers face related to mental health of workers More evidence to properly communicate the issues and inform perspectives Transdisciplinary partnerships with non-OSH professionals to conduct effective work-related mental health studies	A business case to show link between mental health and outcomes of interest Intervention methods that respond to changing definitions of ‘company’ and ‘employee’ Organization-level, rather than individual-level, etiologic and intervention research studies	A compendium of corporate social responsibility lessons to help prioritize well-being as a business issue Examples of success models to help businesses think about new OSH issues Targeted messaging at organizational and policymaker level to have positive impact on mental health A shift in OSH and industry, which recognizes mental health issues and psychosocial exposures as job hazards and outcomes

## Data Availability

Not applicable.

## Appendix A. Workshop Agenda

Southwest Center for Occupational and Environmental Health, UTHealth School of Public Health and CDC/NIOSH

Workshop 2—Virtual Program

*How will the future of work shape research issues, methods and applications? A workshop.*

**Workshop Overview:** This workshop is offered as a part of a multiyear Cooperative Agreement conference grant (U13), in partnership with the CDC/National Institute for Occupational Safety and Health (NIOSH) entitled “Shaping the Future to Ensure Worker Health and Well-being: Shifting Paradigms for Research, Training and Policy.” This virtual workshop is an activity centered on how future changes in the nature of work will (or should) shape occupational safety and health (OSH) research issues, methods, and translation.
The objectives of the workshop are to: 1. : Examine how the future of work will impact OSH research; 2. Identify gaps and needs; 3. Inform the agenda of a larger international conference in 2021, which will address this topic, among others, in the context of an expanded paradigm for OSH.
**Virtual Agenda**

**29 May–3 June 2020:** Opening Address and Theme 1 presentations available online

**Opening Address**

- How the future of work might impact OSH research needs
  John Howard, CDC/NIOSH
- An expanded focus of OSH: a model and research needs
  Paul Schulte, CDC/NIOSH
  **Theme 1: Horizontal Expansion**
- The Future of research on work, safety, health, and well-being
  Glorian Sorensen, Harvard T.H. Chan School of Public Health
  **Theme 1 Ignite Presentations**
- Impact of the changing nature of work on work-family/life issues
  Leslie Hammer, Oregon State University Healthy Workforce Center
- Applications of multi-level analysis to research on work and non-work related factors
  David Gimeno, UTHealth School of Public Health
- Translation research, dissemination and implementation science, & the future of OSH
  Tom Cunningham, CDC/NIOSH
- Cumulative Risk Assessment
  Sudha Pandalai, CDC/NIOSH
- Low wages as determinants of health and research challenges

J. Paul Leigh, University of California at Davis School of Medicine

**4 June 2020: Live moderated session on Theme 1**

This session will include a live, web-based discussion of the Theme 1 presentations, breakout sessions, and a debriefing.

**19 June–24 June 2020: Theme 2 Presentations available online**

**Theme 2: Vertical Expansion**

- Measuring Work and Well-being
  Professor Sir Cary Cooper, CBE University of Manchester
- Research methods applied to the working-life continuum
  Ben Amick, PhD, University of Arkansas for Medical Sciences
  **Theme 2 Ignite Presentations**
- Measurement of well-being
  Ben Miller, PhD, Pardee RAND Graduate School
- Life-course analysis; Mental health
  Ute Bultmann, PhD, University of Groningen (Netherlands)
- Effects of job characteristics on later life cognitive functioning
  Gwen Fisher, PhD, Colorado State University
- Retirement issues and life after work

Carlos Mendes de Leon, PhD, University of Michigan

**25 June 2020: Live moderated session on Theme 2**

This session will include a live, web-based discussion of the Theme 2 presentations, breakout sessions, and a debriefing.

## Appendix B. Workshop Speakers, Participants, and Organizing Committee Members

This appendix provides the names and affiliations of the workshop speakers, participants, and organizing committee.

**Workshop Speakers:**

John Howard, MD, MPH, JD, LLM, MBA (Director, CDC NIOSH, Washington DC 20201, USA)

Paul A. Schulte, PhD (CDC NIOSH, Cincinnati, OH 45226, USA)

Glorian Sorensen, PhD (Harvard T.H. Chan School of Public Health, Boston, MA 02115, USA)

Leslie Hammer, PhD (Oregon State University Healthy Workforce Center, Corvallis, OR 97331, USA)

David Gimeno, PhD (UTHealth School of Public Health, San Antonio, TX 78229, USA)

Tom Cunningham, PhD, (CDC/NIOSH, Cincinnati, OH 45226, USA)

Sudha Pandalai, MD (CDC/NIOSH, Cincinnati, OH 45226, USA)

J. Paul Leigh, PhD (UC Davis Health, Sacramento, CA 95817, USA)

Sir Carey Cooper, CBE (University of Manchester, UK)

Ben Amick, PhD (University of Arkansas for Medical Sciences, Little Rock, AR 72205, USA)

Ben Miller, PhD (Pardee RAND Graduate School, Santa Monica, CA 90407, USA)

Ute Bultmann, PhD (University of Groningen, Groningen, Netherlands)

Gwen Fisher, PhD (Colorado State University, Fort Collins, CO 80523, USA)

Carlos Mendes de León, PhD (University of Michigan, Ann Arbor, MI 48109, USA)

**Workshop Participants:**

Paul Carey, MD (UTHealth School of Public Health, Sugar Land, TX, 77479, USA)

Dawn Castillo, MS, MPH (CDC NIOSH, Morgantown, WV, 26508, USA)

L. Casey Chosewood, MD, MPH (CDC NIOSH, Atlanta, GA, 30329, USA)

Lorraine Conroy, ScD, CIH (University of Illinois at Chicago, Chicago, IL, 60612, USA)

Rena Day, PhD (UTHealth School of Public Health, Houston, TX, 77030, USA)

Rosandra Daywalker, MD (UTHealth School of Public Health, Missouri City, TX, 77459, USA)

Jack Dennerlein, PhD (Harvard T.H. Chan School of Public Health, Dorchester Center, MA, 02124, USA)

Michael Flynn, MA (CDC NIOSH, Cincinnati, OH, 45226, USA)

Ron Goetzel, PhD (Johns Hopkins University IBM Watson Health, Bethesda, MD, 20814, USA)

Rebecca Guerin, PhD (CDC NIOSH, Cincinnati, OH, 45226, USA)

Inkyu Han, PhD (UTHealth School of Public Health, Houston, TX, 77030, USA)

Emily Huang, PhD (Oregon Health & Science University, Portland, OR, 97239, USA)

Doug Johns, MS, PhD (CDC NIOSH, Spokane, WA, 99207, USA)

Erin Kelly, PhD (MIT, Cambridge, MA, 02142, USA)

Niklas Krause, MD, MPH, PhD (University of California Los Angeles, Los Angeles, CA, 90095, USA)

Lee Newman, MD (Colorado School of Public Health, Aurora, CO, 80045, USA)

Heather Padilla, PhD (University of Georgia, Athens, GA, 30602, USA)

Rene Pana-Cryan, PhD (CDC NIOSH, Washington, DC, 20201, USA)

Francisco Perez, PhD (UTHealth School of Public Health, Houston, TX, 77030, USA)

Susan Peters, PhD (Harvard T.H. Chan School of Public Health, Natick, MA, 01760, USA)

John Piacentino, MD, MPH (CDC NIOSH, Washington, DC, 20201, USA)

Frank Pot, PhD (Radboud University, Leiden, 2334 AZ, Netherlands)

Preethi Pratap, PhD (University of Illinois Chicago School of Public Health, Chicago, IL, 60005, USA)

Tapas K Ray, PhD (CDC NIOSH, Cincinnati, OH, 45226, USA)

Rick Rinehart, ScD (The Center for Construction Research and Training, Silver Spring, MD, 20910, USA)

Diane Rohlman, MA, PhD (University of Iowa, Iowa City, IA, 52242, USA)

Natalie Schwatka, PhD, AEP (University of Colorado Anschutz Medical Campus, Aurora, CO, 80045, USA)

William Shaw, PhD (University of Connecticut Health Center, Farmington, CT, 06030, USA)

Gordon Shen, PhD, SM (UTHealth School of Public Health, Houston, TX, 77030, USA)

Julie Sorensen, PhD (Northeast Center for Occupational Health and Safety, Cooperstown, NY, 13820, USA)

Wei-Chung Su, PhD, CIH (UTHealth School of Public Health, Houston, TX, 77030, USA)

Naomi Swanson, PhD (CDC NIOSH, Cincinnati, OH, 45226, USA)

Sara Tamers, PhD, MPH (CDC NIOSH, Washington, DC, 20201, USA)

Dwayne Van Eerd, PhD (Institute for Work & Health, Toronto, Ontario, M5G2E9, Canada)

Gregory Wagner, MD (Harvard T.H. Chan School of Public Health, Boston, MA, 02115, USA)

David Weissman, MD (CDC NIOSH, Morgantown, WV, 26508, USA).

**Organizing Committee (*italics*) and Workshop Staff:**

*George Delclos, MD, MPH, PhD (UTHealth School of Public Health, Houston, TX 77030, USA)*

*Sarah Felknor, MS, DrPH (CDC NIOSH, Atlanta, GA 30333, USA)*

*Paul Schulte, PhD (CDC NIOSH, Cincinnati, OH 45226, USA)*

*Jessica M.K. Streit, PhD, CHES (CDC/NIOSH, Cincinnati, OH 45226, USA)*

*Michelle McDaniel, BS, CHES (UTHealth School of Public Health, Houston, TX 77030, USA)*

Annette Allett (UTHealth School of Public Health, Houston, TX 77030, USA)

Leah Merrill, BBA (UTHealth School of Public Health, Houston, TX 77030, USA)

Silvia Santiago, MAHS (UTHealth School of Public Health, Houston, TX, 77030, USA)

Manal Shafik, MEd (UTHealth School of Public Health, Houston, TX 77030, USA)

## Appendix C. List of Potential Breakout Discussion Questions

Research Needs (gaps that limit the ability of decision-makers (e.g., policymakers, patients, practitioners) from making decisions).

1. What are the major skills that OSH researchers and practitioners will need that they currently lack (to address the expanded OSH paradigm)?
2. Which trends in future employment patterns will influence how OSH research is conducted?
3. How do we establish agreed-upon definitions for various work arrangements in FOW across disciplines that have a vested interest in OSH?
4. How do we effectively balance attention given to new and traditional hazards in FOW?
5. How do we build the evidence base for the interrelationships between work and other life domains to support the expansion of OSH paradigms? (i.e., how do we address the lack of ‘truly TWH’ empirical research in the published literature?)
6. What is the best approach to identify research gaps in an expanded focus for OSH?

Research Methods (strategies, processes or techniques utilized in the collection of data or evidence for analysis in order to uncover new information or create better understanding of a topic.)

1. Which types of surveillance (new or old) will be needed to assess hazards, health effects, and worker well-being?
2. Are there other conceptual approaches to research in the future beside those discussed by the speakers?
3. How should we measure impact of OSH research in the expanded OSH paradigm?
4. How do we design or incorporate OSH research methods and approaches that have a futures orientation?
5. How do we identify/design research measures and metrics that effectively cover the criteria represented by a horizontal expansion of OSH?
6. Given the changing paradigms anticipated in the future of work, what new methods will be needed to fully explore research questions?

Research Translation (knowledge passes along the translational pathway (e.g., research findings translated into practice or policy))

1. How important will implementation science and translation research be in the future?
2. What is the role of translation research and implementation science in an expanded focus for OSH?
3. What new approaches are needed to apply research findings in the future of work?
4. How can OSH funding agencies best support the development of research in an expanded focus for OSH?
5. How do we engage policymakers, practitioners, employers and organized labor in the OSH research process?
